# Catalpol Inhibits Homocysteine-induced Oxidation and Inflammation *via* Inhibiting Nox4/NF-κB and GRP78/PERK Pathways in Human Aorta Endothelial Cells

**DOI:** 10.1007/s10753-018-0873-9

**Published:** 2018-10-12

**Authors:** Huimin Hu, Changyuan Wang, Yue Jin, Qiang Meng, Qi Liu, Zhihao Liu, Kexin Liu, Xiaoyu Liu, Huijun Sun

**Affiliations:** 1 Department of Clinical Pharmacology, College of Pharmacy, Dalian Medical University, 9 West Section, Lvshun South Road, Lvshunkou District, Dalian, 116044 China; 2 Department of Traditional Chinese Medicine, The Second Affiliated Hospital of Dalian Medical University, 467 Zhongshan Road, Shahekou District, Dalian, 116027 China

**Keywords:** catalpol, homocysteine, HAECs, ER stress, Nox4, NF-κB

## Abstract

Hyperhomocysteinemia (HHCY) has been recognized as an independent risk factor for atherosclerosis and plays a vital role in the development of atherosclerosis. Catalpol, an iridoid glucoside extracted from the root of *Rehmannia glutinosa*, can produce anti-inflammatory, anti-oxidant, anti-tumor, and dopaminergic neurons protecting effects. This study aimed to determine the protecting effects of catalpol against homocysteine (HCY)-induced injuries in human aortic endothelial cells (HAECs) and uncover the underlying mechanisms: 1. HAECs were cultured with different concentrations of HCY (3 mM) and catalpol (7.5 μΜ, 15 μΜ, 30 μΜ) for 24 h. (1) The level of MDA and GSH as well as LDH release was measured with colorimetric assay. (2) Reactive oxygen species (ROS) were detected by flow cytometry analysis. (3) Western blotting analysis was performed to detect the expression of Nox4, p22^phox^, ICAM-1, MCP-1, VCAM-1, IκB, nucleus p65, p65 phosphorylation, caspase-3, −9, bax, bcl-2, and ER stress-related proteins. (4) The expressions of CHOP, ATF4 were measured by qRT-PCR. (5) Mitochondrial membrane potential in HCY-treated HAECs was measured by rhodamine 123 staining, and the samples were observed by confocal laser scanning microscopy. 2. DPI, PDTC, and TUDCA were used to determine the interaction among Nox4/ROS, NF-κB, and endoplasmic reticulum stress. 3. TUDCA or Nox4 siRNA were used to investigate whether the effect of catalpol inhibiting the over-production of ROS were associated with inhibiting ER stress and Nox4 expression. Catalpol significantly suppressed LDH release, MDA level, and the reduction of GSH. Catalpol reduced HCY-stimulated ROS over-generation, inhibited the NF-κB transcriptional activation as well as the protein over-expressions of Nox4, ICAM-1, VCAM-1, and MCP-1. Catalpol elevated bcl-2 protein expression and reduced bax, caspase-3, −9 protein expressions in the HCY-treated HAECs. Simultaneously, catalpol could also inhibit the activation of ER stress-associated sensors GRP78, IRE1α, ATF6, P-PERK, P-eIF2α, CHOP, and ATF4 induced by HCY. In addition, the extent of catalpol inhibiting ROS over-generation and NF-κB signaling pathway was reduced after inhibiting Nox4 or ER stress with DPI or TUDCA. The inhibitor of NF-κB PDTC also reduced the effects of catalpol inhibiting the expressions of Nox4 and GRP78. Furthermore, the effect of catalpol inhibiting the over-generation of ROS was reduced by Nox4 siRNA. Catalpol could ameliorate HCY-induced oxidation, cells apoptosis and inflammation in HAECs possibly by inhibiting Nox4/NF-κB and ER stress.

## INTRODUCTION

Endothelial dysfunction is considered as a preliminary event of pathophysiologic importance in the development of atherosclerosis (AS) [1, 2], and provides a significant link between diseases, including hypertension, diabetes, and other high-risk cardiovascular diseases. Homocysteine (HCY) is an intermediate [3], derived from sulfur-containing amino acid metabolism. Elevated level of circulating HCY, called hyperhomocysteinemia (HHCY), is a common and an independent risk factor for atherogenesis and diabetic cardiovascular diseases (CVDs) [4–7]. Previous studies showed that HCY-induced endothelial dysfunction is associated with oxidative stress and ER stress, probably due to stimulating inflammatory response as well as causing disturbance in the anti-thrombotic activities of the endothelium [8].

Pieces of evidence indicate that HHCY is linked with increased risk of oxidative stress injury. The pathophysiological level of HCY is implicated to reduce bioavailability of NO, accelerate superoxide and peroxynitrite generation, and inhibit anti-oxidant defense in cardiovascular system [9–11]. Reactive oxygen species (ROS) have been identified as mediators in the thiol of HCY auto-oxidation [12]. Therefore, administration of anti-oxidant is widely involved in attenuating the impairment of cardiovascular function induced by HHCY.

The endoplasmic reticulum (ER) and mitochondria are viewed as important cellular organelles to preserve cellular homeostasis, and ER and mitochondria interact with each other physically and functionally [13]. Recent studies suggested that HCY could trigger ER stress by perturbing disulfide bond formation and inducing unfolded protein response (UPR) [14]. One crucial response of UPR signaling is to elevate protein synthesis to perturb homeostasis, such as PERK, eIF2α, and IRE1α. The other is to activate a coordinately regulated transcriptional network, including ATF4, ATF6, and XBP-1 [15]. Simultaneously, HCY could activate ER-mitochondria coupling and mitochondrial metabolic reprogramming, containing mitochondrial ROS generation, calcium signal and membrane potential [16].

Catalpol is an iridoid glycoside extracted from the fresh roots of *radix rehmannia*. It has been demonstrated to exert anti-inflammatory, anti-oxidant, anti-apoptotic, and other neuroprotective properties, and has a role in neuroprotection against ischemic injuries and neurodegenerative diseases. Catalpol can reduce oxidative damage, regulate endocrine function, and enhance anti-inflammatory properties [17]. Catalpol can also protect against cardiovascular injuries by attenuating free radicals, lipid peroxidation, and cell apoptosis [18]. However, the underlying mechanism of catalpol on endothelial dysfunction induced by HCY in HAECs has not been fully elucidated. In the current study, we investigated the causative role of catalpol on HCY-induced injuries in HAECs. Furthermore, we also analyzed the involvement of Nox4/NF-κB and GRP78/PERK pathways in mediating the protecting effects of catalpol in HAECs.

## MATERIALS AND METHOD

### Reagent and Antibodies

Human aorta endothelial cells (HAECs) were purchased from Shanghai Bioleaf Biotech Co, Ltd. (Shanghai, China). Cell culture media (DMEM) and fetal bovine serum (FBS) were obtained from the Gibco-BRP Company (Gaithersburg, MD, USA). Catalpol (at a purity of ≥ 99.5%) was bought from Nanjing Jingzhu Biotechnology Co., Ltd. (Jiangsu, China). DL-homocysteine (HCY) was purchased from Sigma-Aldrich (St Louis, MO, USA). DCFH-DA fluorescent probe and ECL Plus were purchased from Beyotime Institute of Biotechnology (Jiangsu, China). Rabbit anti-human polyclonal antibodies recognizing Nox4, p65, bcl-2, cleaved caspase-3, caspase-9, cytochrome c, ATF6, CHOP, and IκB polyclonal antibodies were obtained from Proteintech Group, Inc. Rabbit anti-GRP78 polyclonal antibody was purchased from Wuhan Boster Bio-Engineering Limited Company. Rabbit anti-PERK antibody and rabbit anti-phospho-PERK were purchased from Beijing Biosynthesis Biotechnology Co., Ltd. Polyclonal antibodies specific for eIF2α, phospho-eIF2α were purchased from Beyotime Biotechnology (Jiangsu, China). Antibodies specific for IRE1, phosph-IRE1 (phosphor S724) were purchased from Abcam (Hong Kong) Ltd. Mouse anti-beta actin monoclonal antibody and gold anti-rabbit IgG-HRP were purchased from Zhongshan Golden Bridge Biotechnology Co., Ltd. (Beijing, China).

### Cell Culture Studies

HAECs were cultured in DMEM medium supplemented with 10% fetal bovine serum (FBS), 1 U/mL penicillin, and 100 μg/mL streptomycin. Cells were incubated at 37 °C in humidified atmosphere with 5% CO_2._ The cultured cells were made quiescent by incubation with serum-free basal medium for 24 h before use.

### SOD, MDA, GSH, and LDH Assays

In order to estimate the damage level of endothelial cells, we applied a colorimetric assay kit (Beyotime, Nanjing, China) to measure malonaldehyde (MDA), glutathione (GSH), and lactate dehydrogenase (LDH). In summary, HAECs were incubated in 6-well plates at a density of 1 × 10^5^ each well. The cells were then cultivated for 24 h with different concentrations of catalpol (7.5, 15, 30 μM) and HCY (3 mM) for 24 h.

### Measurement of Intracellular ROS

ROS level was measured by the fluorescent probe, 2′, 7′-dichlorodihydrofluorescein diacetate (DCFH-DA) probe as previously described [19]. HAECs were cultured in 6-well plates at a density of 2 × 10^5^ per well, while the cells were incubated with different concentrations of catalpol for 23 h at 37 °C, HCY was followed for 1 h. At the end of treatment, the culture medium was changed to serum-free medium with DCFH-DA (10 μM) at 37 °C. After 30 min, the fluorescence intensity was quantified by a BD FACSCalibur Flow Cytometer (Becton, Dickinson and Company, USA).

### Fluorescence Microscopy

HAECs were seeded in 6-well plates at a density of 1 × 10^5^/well. The cells were treated with catalpol (7.5, 15, and 30 μM) and HCY (3 mM) at 37 °C for 24 h. At the end of treatment, the cells were fixed with 4% paraformaldehyde for 15 min, rinsed with PBS, and mounted in mounting medium with DAPI. The samples were observed by an inverted fluorescence microscope.

### Terminal Deoxynucleotide Transferase dot Nick-End Labeling (TUNEL) Assay

Apoptosis was measured by TUNEL assay using TUNEL apoptosis detection kit (Bio tool, Houston, TX, USA). Briefly, after treatment with different concentrations of catalpol (7.5, 15, 30 μM) for 24 h, the cells were fixed with 4% paraformaldehyde for 15 min and rinsed with PBS. After permeabilization with 0.5% tween 20, HAECs were incubated at room temperature for 1 h in a moist chamber with TUNEL mixture as recommended by the manufacturer. Then, the samples were observed by an inverted fluorescence microscope.

### Measurement of Mitochondrial Transmembrane Potential

Changes in mitochondrial membrane potential were detected in the presence of the fluorescent rhodamine 123 (rh123). After treatment with different concentrations of catalpol (7.5, 15, 30 μM) for 24 h, the HAECs were cultivated with rh123 (1 mg ml^−1^ in dimethyl sulfoxide) at 37 °C for 1 h and rinsed with PBS. The cells were collected and analyzed by confocal laser scanning microscope (Leica microsystems).

### RNA Isolation and Real-Time RT-PCR Analysis

The transcript level of cells damage was determined in the cDNA sample by using quantitative real-time PCR. Total RNA was extracted with RNAiso plus reagent following the manufacturer’s instructions (Takara Biotechnology, Dalian, China) and cDNA was synthesized with the Primescript RT reagent Kit with gDNA Eraser (Takara Biotechnology, Dalian, China). Then, the cDNA was supplemented with primers and PCR-mix according to the manufacturers’ protocol. Real-time PCR amplification and detection were performed using the ABI PRISM 7500 real-time PCR system (Applied Biosystems, USA) with the SYBR Premix EX Taq™. Relative mRNA amount was calculated by the 2^(−delta delta CT)^ method using β-actin as an internal control for each sample.

The primer sequences for GRP78:

- Forward: 5′-CACAGTGGTGCCTACCAAGAAG-3′
- Reverse: 5′-AGCAGGAGGAATTCCAGTCAGA-3′
- The primer sequences for CHOP:
- Forward: 5′-GCTTCTCTGGCTTGGCTGACT-3′
- Reverse: 5′-CTGTTTCCGTTTCCTGGTTCTC-3′
- The primer sequences for ATF4:
- Forward: 5′-CCAGCAAAGCACCGCAACA-3′
- Reverse: 5′-CCATCCACAGCCAGCCATT-3′
- The primer sequences for β-actin:
- Forward: 5′-TGGAACCCAGCACAATGAA-3′
- Reverse: 5′-CTAAGTCATAGTCCGCCTAG-3′

### Western Blotting Analysis

The samples were homogenized in a whole cell extract buffer. Equal amounts of protein was resolved on a SDS-PAGE (8%, 10%, 15%) and transferred onto polyvinyl difluoride (PVDF) membranes. The reacted band was visualized by chemiluminescence (ECL plus, Beyotime Institute of Biotechnology, Shanghai, China). The blots were analyzed with Gel-Pro analyzer software according to manufacturer’s instruction. To account for possible difference in the protein load, the density of each band was divided by the density of the respective of β-actin.

### siRNA Transfection

siRNA against Nox4 was purchased from Life Technologies (CA, USA) for transfection. The sense and anti-sense strands of the Nox4 siRNA were 5′-CCAUGUGCCGAACACUCUUTT-3′ and 5′-AAGAGUGUUCGGCACAUGGT-3′, respectively. The siRNA transfection was performed with Lipofectamine™ 2000 (Life Technologies, CA, USA), according to the manufacture’s guide.

### Statistics

The results were collected and analyzed using SPSS software package (version 19.0, SPSS), and all of the values were expressed as the mean ± SD. Comparison of quantitative variables was performed by ANOVA followed by the Student-Newman-Keuls (SNK) test. *P*-values < 0.05 (two-tailed) were considered statistically significant.

## RESULT

### Catalpol Decreased the Level of MDA and LDH Release as well as Increased GSH Generation in HCY-Treated HAECs

**Fig. 1:**
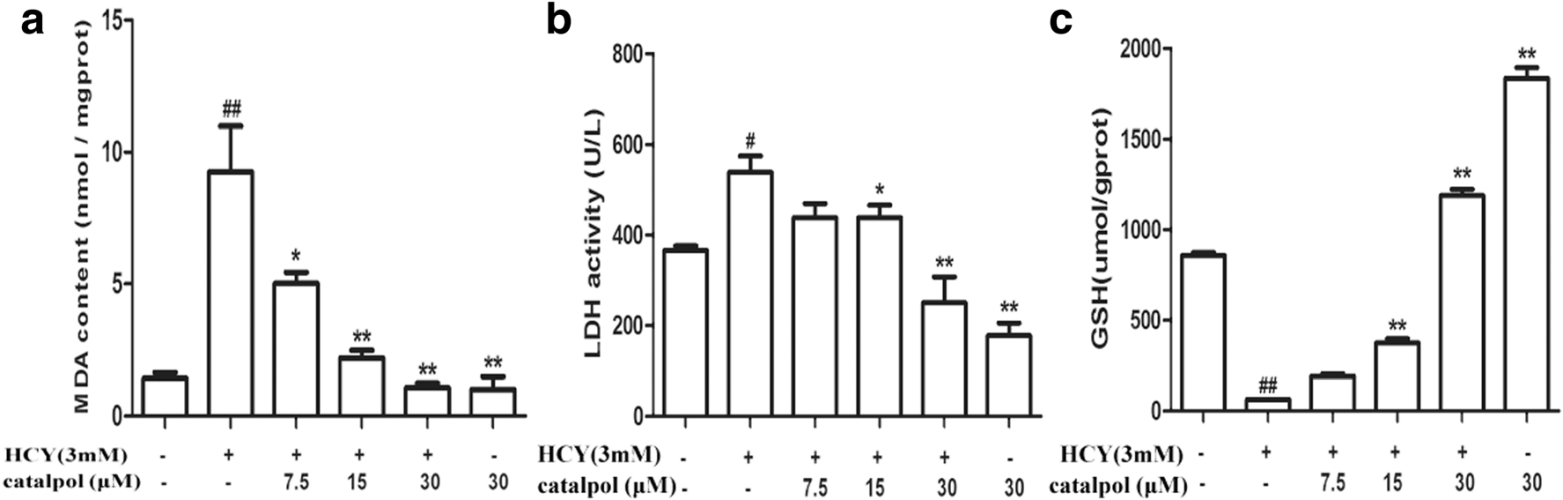
Effects of catalpol on MDA level, LDH release, and GSH level induced by HCY in HAECs. (**a**) Intracellular level of MDA. (**b**) The release of LDH. (**c**) The level of GSH. Data are expressed as means ± S.D. from three independent experiments. ^*#*^*p* < 0.05 *vs.* control, ^*^p < 0.05 *vs.* HCY, ^*##*^*p* < 0.01 *vs.* control, ^**^p < 0.01 *vs.* HCY.

MDA is the final product of oxygen radicals, which is a marker of lipid peroxidation [20]. LDH is considered as an indicator of irreversible cell death that leaked from cells after plasma membrane disruption. As demonstrated in Fig. 1a, b, the increased levels of MDA and LDH induced by HCY were significantly reduced by catalpol. GSH, as a co-factor for glutathione peroxidase and the redox enzyme, catalyzes the reduction of lipid peroxidation [21]. The GSH redox cycle involves in ROS scavenging and is considered as the most important anti-oxidant system in endothelial cells. As showed in Fig. 1c, HCY could significantly elevate depletion of cellular GSH; however, catalpol efficiently reduced the GSH depletion in HAECs dose-dependently.

### Catalpol Attenuated HCY-Induced ROS over-Generation and NADPH Oxidase over-Expression

**Fig. 2:**
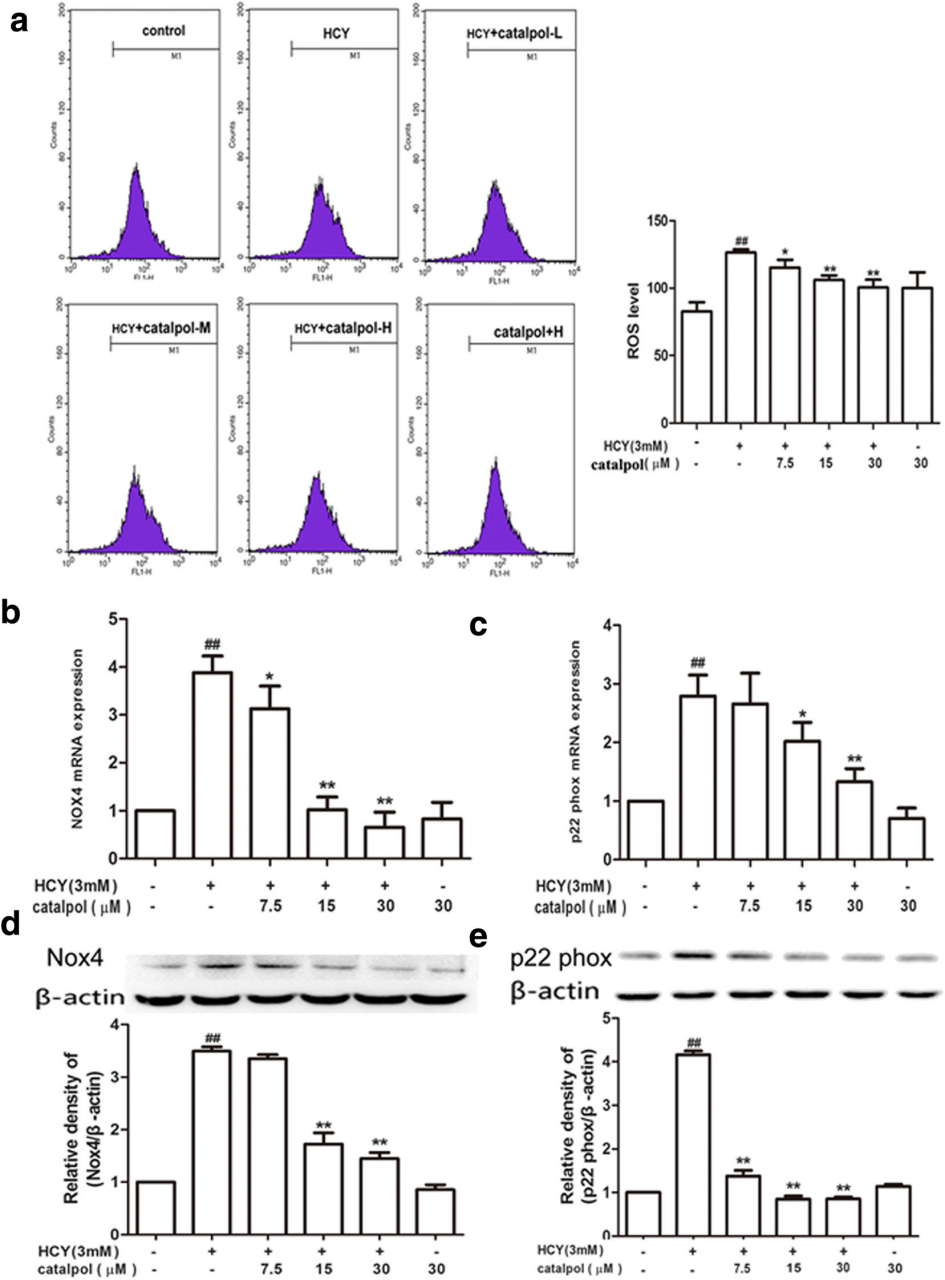
Effects of catalpol on ROS over-production and NADPH oxidase over-expression induced by HCY. (**a**) Effect of catalpol on ROS generation. (**b**) Effect of catalpol on Nox4 mRNA expression level. (**c**) Effect of catalpol on Nox4 protein expression level. (**d**) Effect of catalpol on p22^phox^ mRNA expression level. (**e**) Effect of catalpol on p22^phox^ protein expression level. Data are expressed as means ± S.D. from three independent experiments. ^*^p < 0.05 *vs.* HCY, ^*##*^*p* < 0.01 *vs.* control, ^**^p < 0.01 *vs.* HCY.

ROS generated from mitochondrial respiration caused oxidative damage to nucleic acid, lipids, carbohydrates, and proteins [22]. Intracellular ROS were detected by using the ROS indicator DCFH-DA as a probe. As demonstrated in Fig. 2a, the DCF-fluorescence in the HCY-induced cells was substantially increased compared with the control group. After catalpol treatment, the fluorescence was strikingly attenuated, indicating that catalpol could reduce ROS generation. NADPH oxidase enzymes are the primary sources of vascular ROS, with Nox4 playing the key role in vasculopathies and p22^phox^ is a modulatory isoform for NADPH oxidase [23]. Thus, the effects of catalpol on HCY-induced Nox4 and p22^phox^ mRNA and protein expressions were examined. Compared with the responses in control group, HCY obviously elevated the mRNA and protein expressions of Nox4 and p22^phox^. In contrast, catalpol could significantly inhibit the elevation in levels of Nox4 and p22^phox^ protein expressions (Fig. 2a, b, c, d).

### Catalpol Ameliorated HCY-Induced ROS over-Generation *via* Inhibiting Nox4 Activation in HAECs

**Fig. 3:**
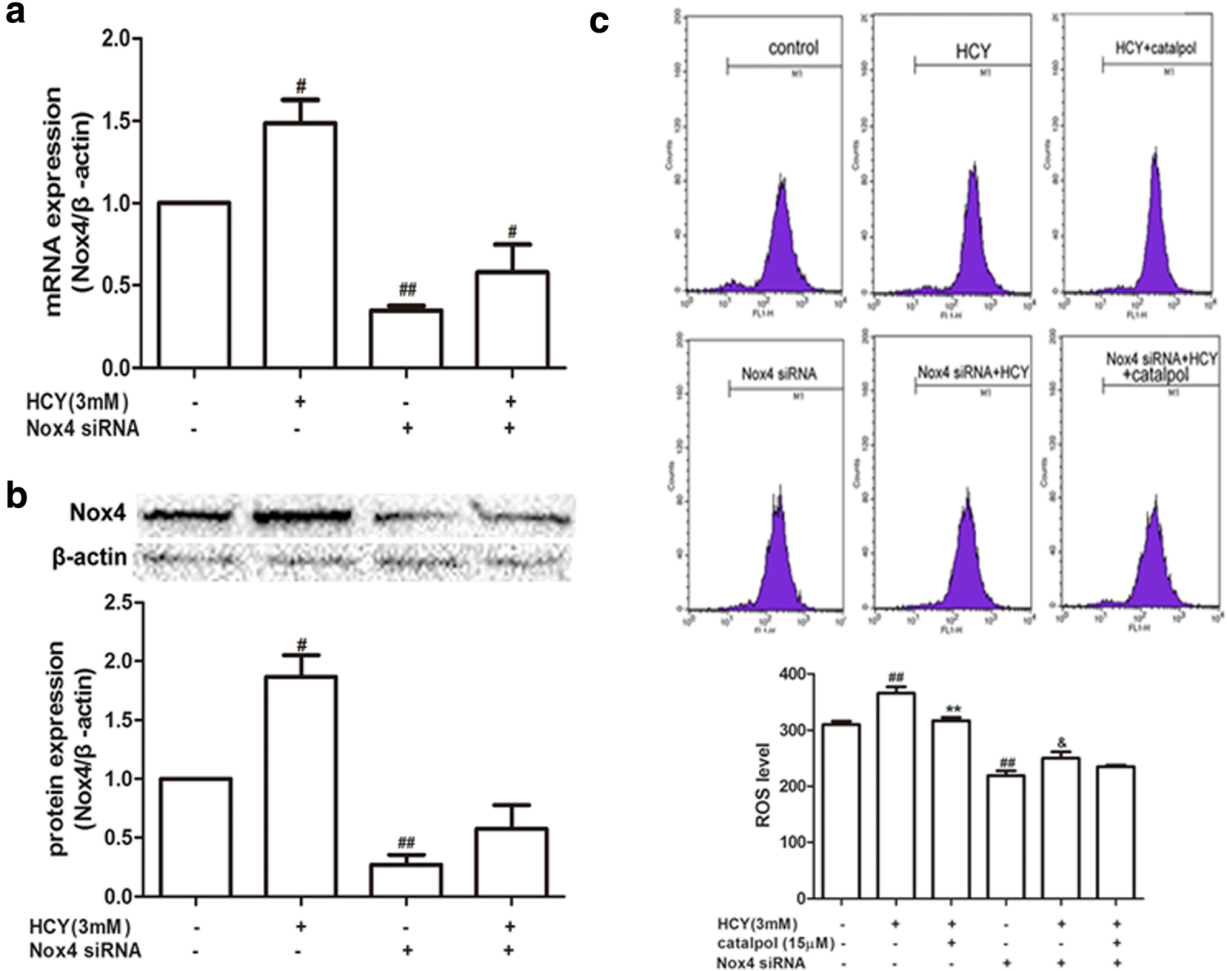
Effect of catalpol on ROS over-generation induced by HCY in normal and Nox4 knock-down cells. Cells were transfected with or without Nox4 siRNA (50 nM) for 6 h and incubated for 24 h, followed by treatment with catalpol (25 μΜ) then incubation with HCY (3 mM) for 2 h. (**a**) Nox4 mRNA expression. (**b**) Nox4 protein expression. (**c**) The intracelluar ROS production. Data are expressed as means ± S.D. from three independent experiments. ^*#*^*p* < 0.05 *vs.* control, ^##^*p* < 0.01 *vs.* control, ^**^p < 0.01 *vs.* HCY, ^*&*^*p* < 0.01 *vs.* Nox4 siRNA.

In order to further investigate whether catalpol reduced ROS generation was involved in inhibiting Nox4 expression, we employed siRNA to Nox4 and then estimated HCY-induced ROS generation. As showed in Fig. 3a, b, the Nox4 expressions of mRNA and protein were remarkably inhibited and it confirmed that Nox4 was knocked down definitely. In normal group, HCY exposure significantly increased ROS generation in HAECs. After treated with catalpol, ROS generation level was markedly decreased as compared to the HCY-induced group. However, there was no significant change in ROS generation after treated with catalpol in Nox4 knocked-down cells (Fig. 3c). It suggested that catalpol reduced HCY-induced ROS over-generation possibly *via* blocking Nox4 signaling pathway.

### Catalpol Down-Regulated HCY-Induced Inflammatory Response in HAECs

**Fig. 4:**
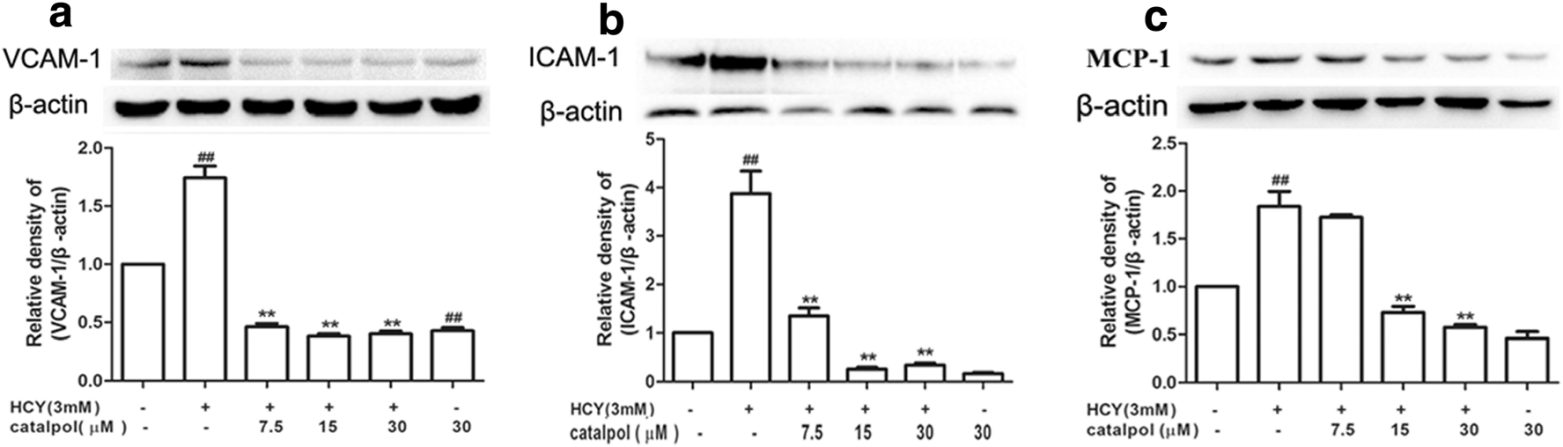
Effects of catalpol on the protein expressions of adhesion molecules in HCY-stimulated HAECs. (**a**) Effect of catalpol on VCAM-1 protein expression. (**b**) Effect of catalpol on ICAM-1 protein expression. (**c**) Effect of catalpol on MCP-1 protein expression. Data are expressed as means ± S.D. from three independent experiments. ^*##*^*p* < 0.01 *vs.* control, ^**^p < 0.01 *vs.* HCY.

Homocysteine-induced inflammation within the endothelial cells plays a pivotal role on the etiology and pathogenesis of atherosclerosis. In order to further investigate the inhibitory effects of catalpol on the production of pro-inflammatory cytokines in HCY-induced cells, we measured the protein expressions of ICAM-1, VCAM-1, and MCP-1, which reflect the activation of inflammatory response. The results showed that the protein levels of ICAM-1, VCAM-1, and MCP-1 were all significantly elevated in HCY-treated cells. However, catalpol could cause significant reduction in the protein expression levels of ICAM-1, VCAM-1, and MCP-1 compared to HCY-treated group (Fig. 4a, b, c). These findings demonstrated that catalpol could protect HAECs from HCY-induced inflammatory responses to safeguard endothelial function.

### Catalpol Inhibited HCY-Induced Apoptosis of HAECs

**Fig. 5:**
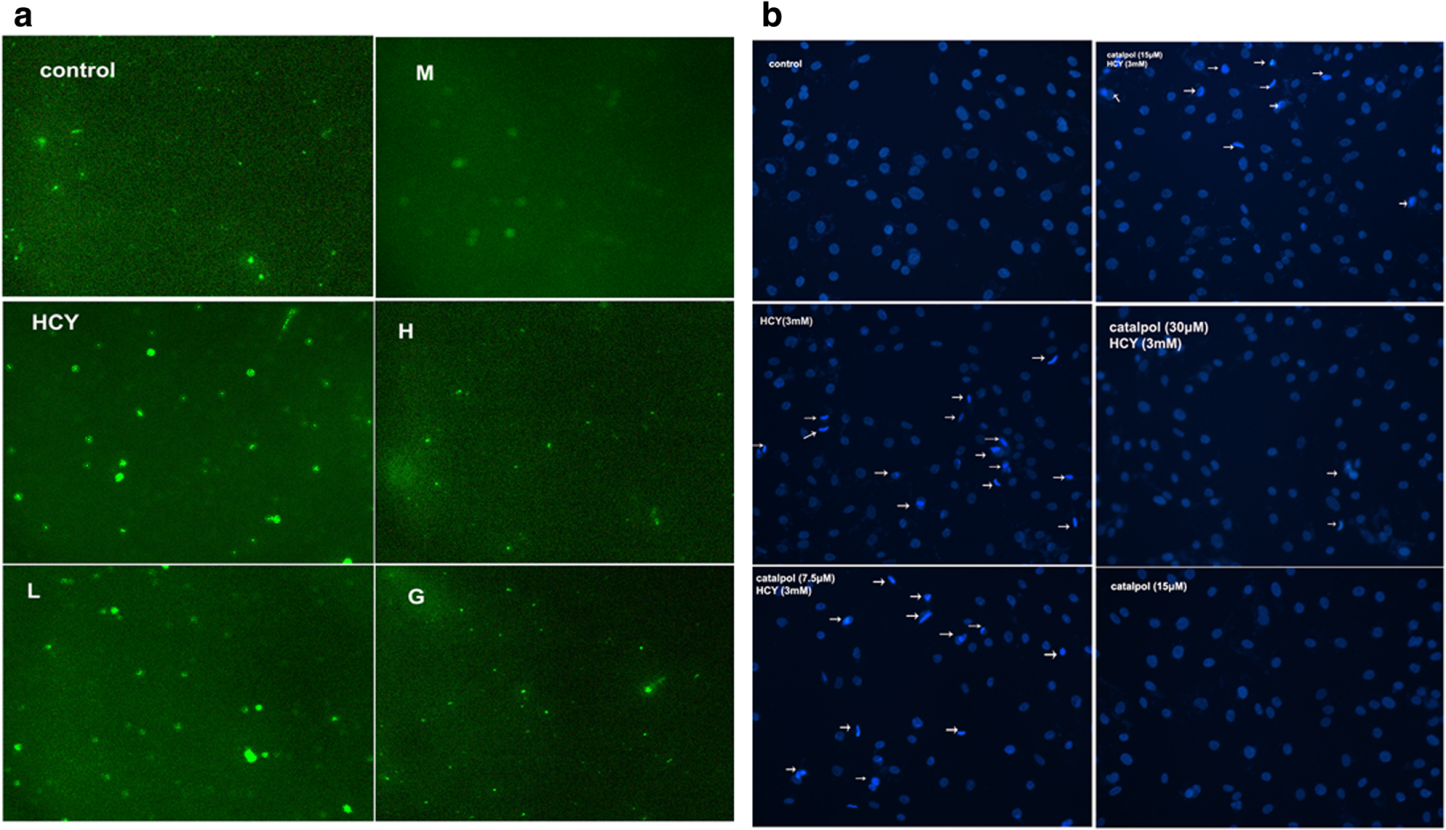
Catalpol inhibited HCY-induced apoptosis of HAECs. Cells were treated with different concentrations of catalpol (7.5, 15, 30 μM) and HCY (3 mM) for 24 h. (**a**) Fluorescence microscopy after labeling of treated cells with TUNEL staining (200×). (**b**) Fluorescence microscopy after labeling of treated cells with DAPI (1 μg/ml) staining (200×).

The apoptosis of endothelial cells is considered to play a critical role in the pathological mechanism of cardiovascular diseases, which was associated with HHCY [24]. To investigate the anti-apoptotic effects of catalpol, the TUNEL assay was performed. As shown in Fig. 5a, the quantities of TUNEL-apoptosis cells were markedly increased in HCY-induced group compared with the control group. However, in catalpol-treated groups, the positive rates of TUNEL-apoptosis cells were significantly attenuated. Consistent with the result measured by TUNEL assay, HCY induced the appearance of more DAPI-positive cells, which exhibited the morphological features of heterogeneous intensity and chromatin condensation of nuclei. In contrast, catalpol strikingly inhibited the cell apoptosis induced by HCY (Fig. 5b).

### Catalpol Improved the Expression of bcl-2 and Inhibited the Protein Levels of bax, Cleaved Caspase-3, Caspase-9, and Cytochrome c

**Fig. 6:**
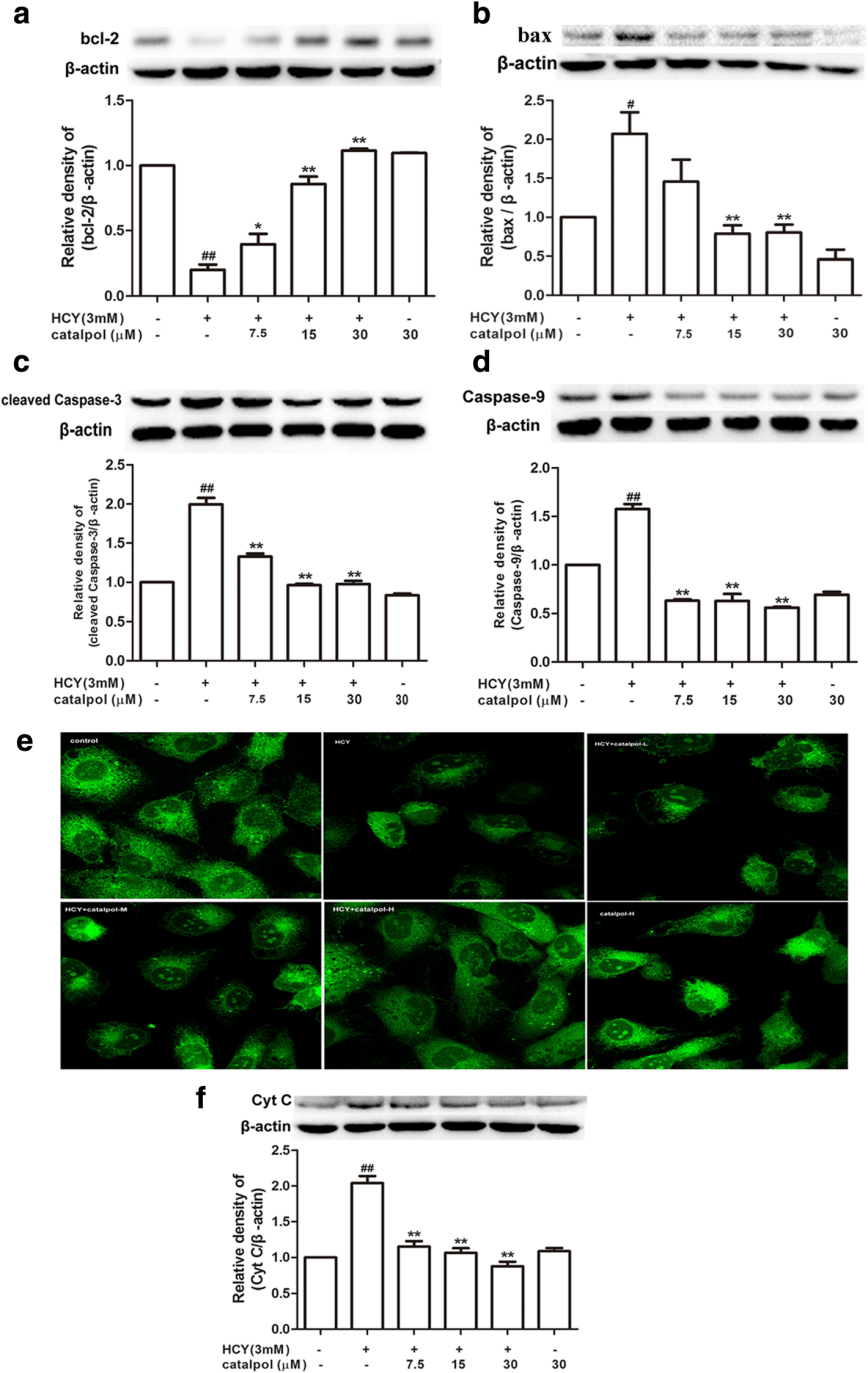
Catalpol enhanced the protein expression of bcl-2 and reduced the protein expressions of bax, cleaved caspase-3 and caspase-9 as well as cytochrome c. (**a**) Effect of catalpol on bcl-2 protein expression level. (**b**) Effect of catalpol on bax protein expression level. (**c**) Effect of catalpol on cleaved caspase-3 protein expression leve. (**d**) Effect of catalpol on caspase-9 protein expression level. (E) Effect of catalpol on mitochondrial transmembrane potential (Ψm). Confocal laser scanning microscopy after rhodamine 123 staining, HAECs were cultured with HCY (3 mM) and catalpol (7.5, 15, 30 μM). (**f**) Effect of catalpol on cytochrome c protein expression level. Results represent the mean ± S.D. from three independent experiments. ^*#*^*p* < 0.05 *vs.* control, ^*^p < 0.05 *vs.* HCY, ^*##*^*p* < 0.01 *vs.* control, ^**^p < 0.01 *vs.* HCY.

It is well known that the programmed cell death of numerous cell types is implicated with the caspase family of cysteine proteases. Thus, we investigated the activation of cleaved caspase-3, −9 in HCY-treated cells by western blotting. The expression levels of cleaved caspase-3 and caspase-9 were significantly increased in HCY-induced group compared to the control group (Fig. 6c, d). Moreover, catalpol clearly attenuated this phenomenon. Additionally, bcl-2 family proteins, including bcl-2 and bax, are widely involved in regulating the intrinsic or mitochondrial apoptotic pathway. It was also showed that HCY decreased the protein expression of anti-apoptotic protein bcl-2 and increased the level of pro-apoptotic protein bax. In catalpol treatment groups, the expressions were altered obviously (Fig. 6a, b). Cytochrome c plays a pivotal role on programmed cell death, acting as a main apoptotic protease-activating factor secreted by mitochondria. As demonstrated in Fig. 6f, cytochrome c release was elevated in HCY-induced group. After catalpol treatment, the elevation was significantly attenuated. The mitochondrial membrane potential (Ψm) is considered as a key indicator of mitochondrial function and is involved in the initial process of cell apoptosis cascade reaction and rhodamine 123 fluorescence is a vital monitor to evaluate the mitochondrial membrane potential [25]. As shown in Fig. 6e, a collapse of Ψm could be observed in HCY-stimulated HAECs. Nevertheless, after treatment with catalpol, the Ψm was obviously enhanced in a concentration-dependent manner. Taken together, these results indicated that catalpol could produce obvious anti-apoptotic effects in HAECs treated by HCY.

### Catalpol Protected Against ER Stress-Mediated Apoptotic Processes in HCY-Treated HAECs

**Fig. 7:**
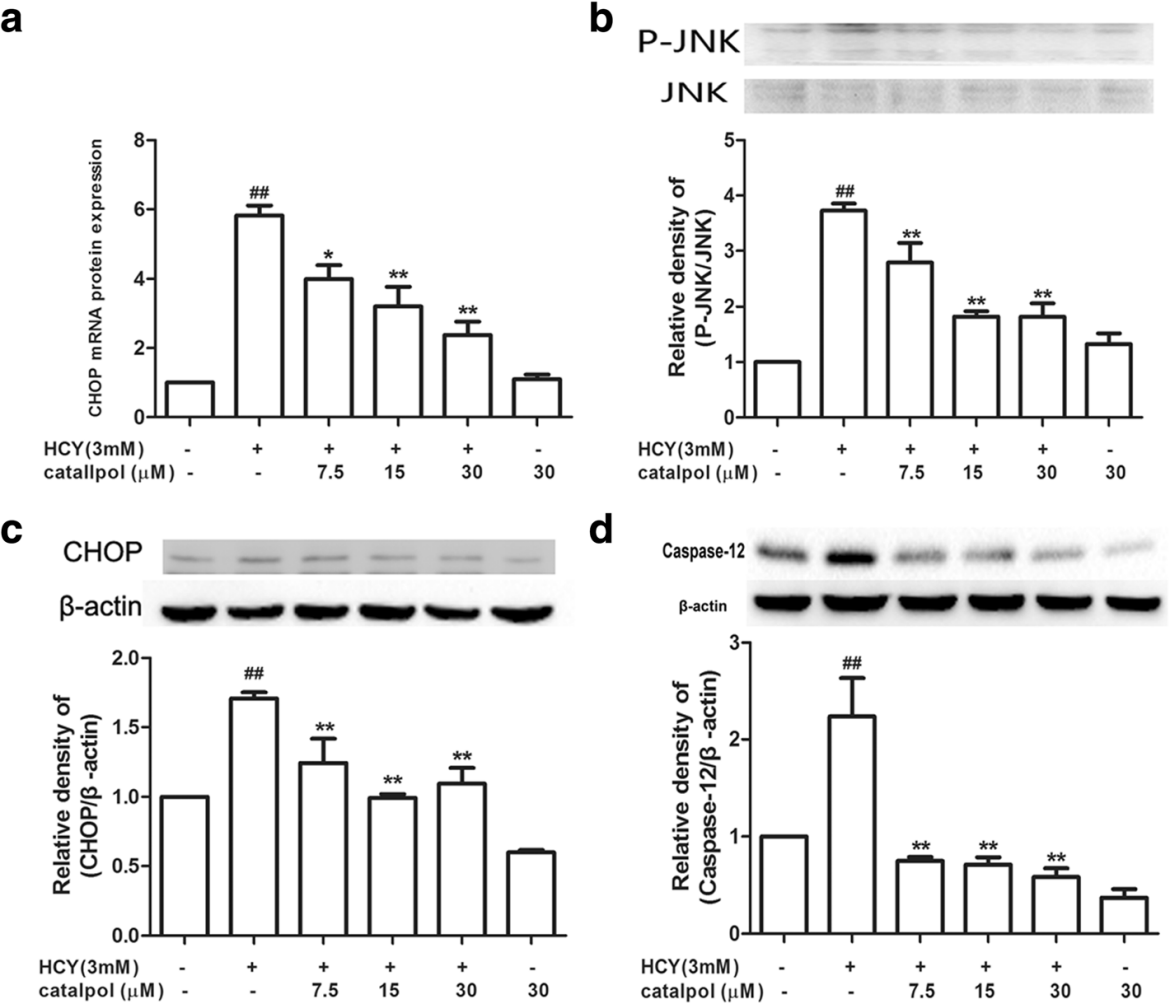
Catalpol inhibited HCY-induced ER stress-associated apoptosis of HAECs. Cells were treated with different concentrations of catalpol (7.5, 15, 30 μM) and HCY(3 mM) for 24 h. (**a**) Effect of catalpol on CHOP mRNA expression level. (**b**) Effect of catalpol on P-JNK protein expression level. (**c**) Effect of catalpol on CHOP protein expression level. (**d**) Effect of catalpol on caspase-12 protein expression level. Results represent the mean ± S.D. from three independent experiments. ^*^p < 0.05 *vs.* HCY, ^*##*^*p* < 0.01 *vs.* control, ^**^p < 0.01 *vs.* HCY.

It is well known that prolong and severe ER stress results in cell apoptosis, which is mediated by CHOP, JNK, and caspase-12 [26]. To further confirm whether catalpol defended against ER stress-induced apoptosis, we primarily detected HCY-induced CHOP mRNA and protein expressions. As shown in Fig. 7a, c, compared to the HCY treatment alone group, the mRNA and protein expressions of CHOP were significantly reduced in catalpol treatment groups. Upon the induction of ER stress, IRE1 kinase becomes capable of recruiting tumor necrosis factor receptor-associated factor (TRAF2), which couples a plasma-membrane death receptor to JNK [27]. As demonstrated in Fig. 7b, HCY obviously activated the expression of phosphorylated JNK, whereas, catalpol could cause a significant decrease in the level of phosphorylated JNK. Caspase-12, localizing on the ER cytoplasmic side, is activated specifically by ER stress [26]. Consistent with CHOP and JNK expressions, catalpol could notably alleviate the increase of caspase-12 protein expression induced by HCY (Fig. 7d). Thus, catalpol could ameliorate ER stress-mediated cell apoptosis.

### Catalpol Relieved the Over-Expression and Activation of ER Stress-Associated Marker Proteins in HAECs Induced by HCY

**Fig. 8:**
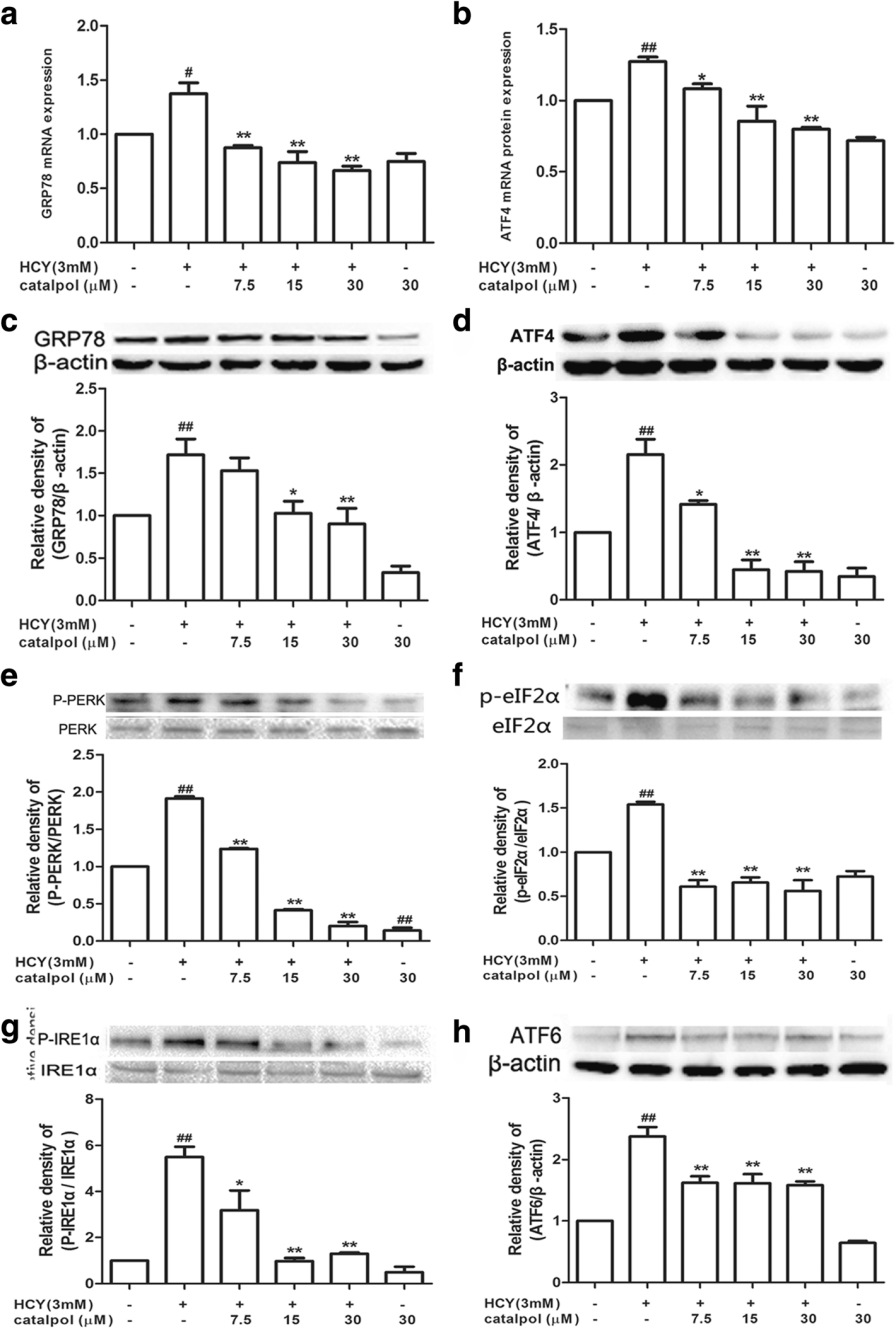
Catalpol inhibited HCY-induced ER stress in HAECs. (**a**) Effect of catalpol on GRP78 mRNA expression level induced by HCY. (**b**) Effect of catalpol on ATF4 mRNA expression level induced by HCY. (**c**) Effect of catalpol on GRP78 protein expression level induced by HCY. (**d**) Effect of catalpol on ATF4 protein expression level induced by HCY. (**e**) Effect of catalpol on P-PERK protein expression level induced by HCY. (**f**) Effect of catalpol on P-eIF2α protein expression level induced by HCY. (**g**) Effect of catalpol on P-IRE1α protein expression level induced by HCY. (**h**) Effect of catalpol on ATF6 protein expression level induced by HCY. Results represent the mean ± S.D. from three independent experiments. ^*#*^*p* < 0.05 *vs.* control, ^*^p < 0.05 *vs.* HCY, ^*##*^*p* < 0.01 *vs.* control, ^**^p < 0.01 *vs.* HCY.

To confirm the effect of catalpol on ER stress induced by HCY in HAECs, the expressions of ER stress-associated markers were observed by using Western blotting and real-time RT-PCR. Results from Fig. 8a, c showed that the mRNA and protein levels of the molecular chaperone GRP78 were dramatically activated in HCY-induced cells compared to the control cells. However, the administration of catalpol suppressed the activation. Consistent with GRP78, the protein expressions of other three ER stress sensors, PERK, ATF6, and IRE1α, were also significantly increased in HCY-induced HAECs and catalpol could decline the over-expressions (Fig. 8e, g, h). Then, we further detected other ER stress-associated indicators. As shown in Fig. 8b, d, f, the accumulation of phosphorylated PERK-activated its downstream sensor, phosphorylated eIF2α, which increased the mRNA and protein expressions of ATF4 in HCY-induced HAECs. However, catalpol contributed to relieve this process in endothelial cells. The results demonstrated that catalpol could inhibit the over-expressions of ER stress-related proteins.

### Catalpol Inhibited the Degradation of IκB and Transcriptional Activation of NF-κB Induced by HCY

**Fig. 9:**
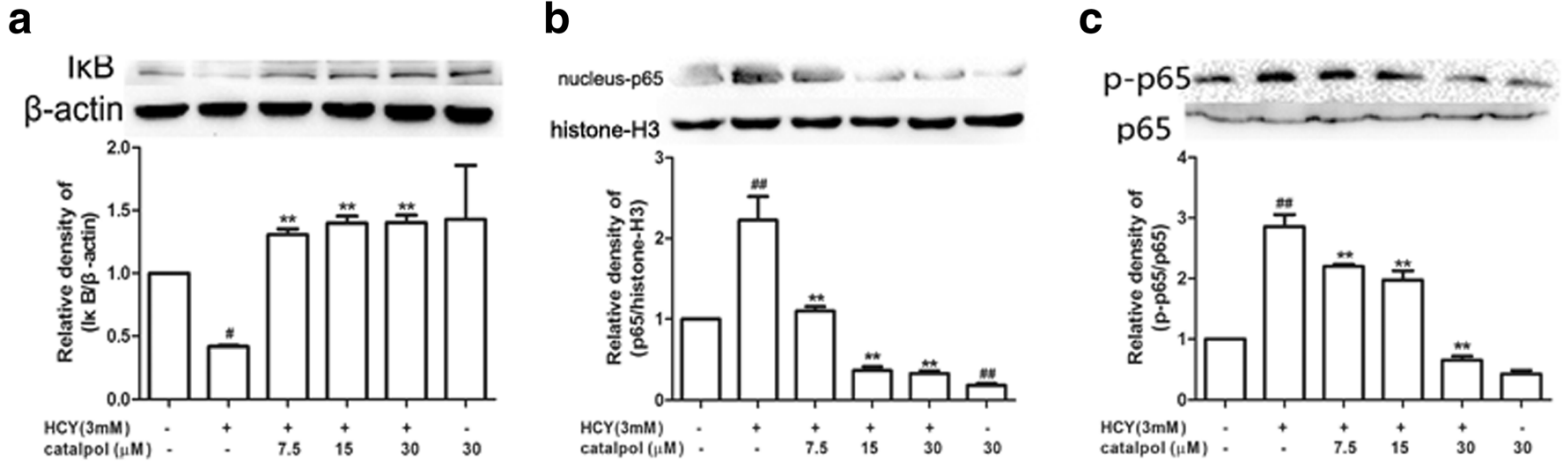
Effect of catalpol on NF-κB pathway activation induced by HCY in HAECs. (**a**) Effect of catalpol on IκB protein expression level induced by HCY. (**b**) Effect of catalpol on nucleus p65 protein expression level induced by HCY. (**c**) Effect of catalpol on the phosphorylation level of p65. Results represent the mean ± S.D. from three independent experiments. ^*#*^*p* < 0.05 *vs.* control, ^*##*^*p* < 0.01 *vs.* control, ^**^p < 0.01 *vs.* HCY.

NF-κB has long been considered as a proatherogenic factor in multiple pathological and physiological processes during atherogenesis [28]. The activation of NF-κB pathway might be induced by reduced IκB expression in cytosol degradation. Therefore, we primarily measured the effect of catalpol on the expression of IκB in HCY-induced HAECs. As demonstrated in Fig. 9a, in HCY-stimulated cells, the protein level of IκB was significantly decreased. However, after treatment with catalpol, the protein expression of IκB was typically increased. Subsequently, NF-κB/p65 nuclear translocation was detected by Western blotting. The results showed that HCY typically activated the expressions of phosphorylated p65 and induced the translocation of p65 in the nucleus. Catalpol could partly inhibit HCY-induced expressions of phosphorylated p65 and translocation of p65 (Fig. 9b, c). Collectively, these results showed that catalpol could significantly inhibit the degradation of IκB, expressions of phosphorylated p65 and translocation of p65, in turn, could inhibit the activation of NF-κB signaling pathway.

### Catalpol Attenuated HCY-Induced Activation of NF-κB Pathway and ER Stress *via* Inhibiting Nox4 in HAECs

**Fig. 10:**
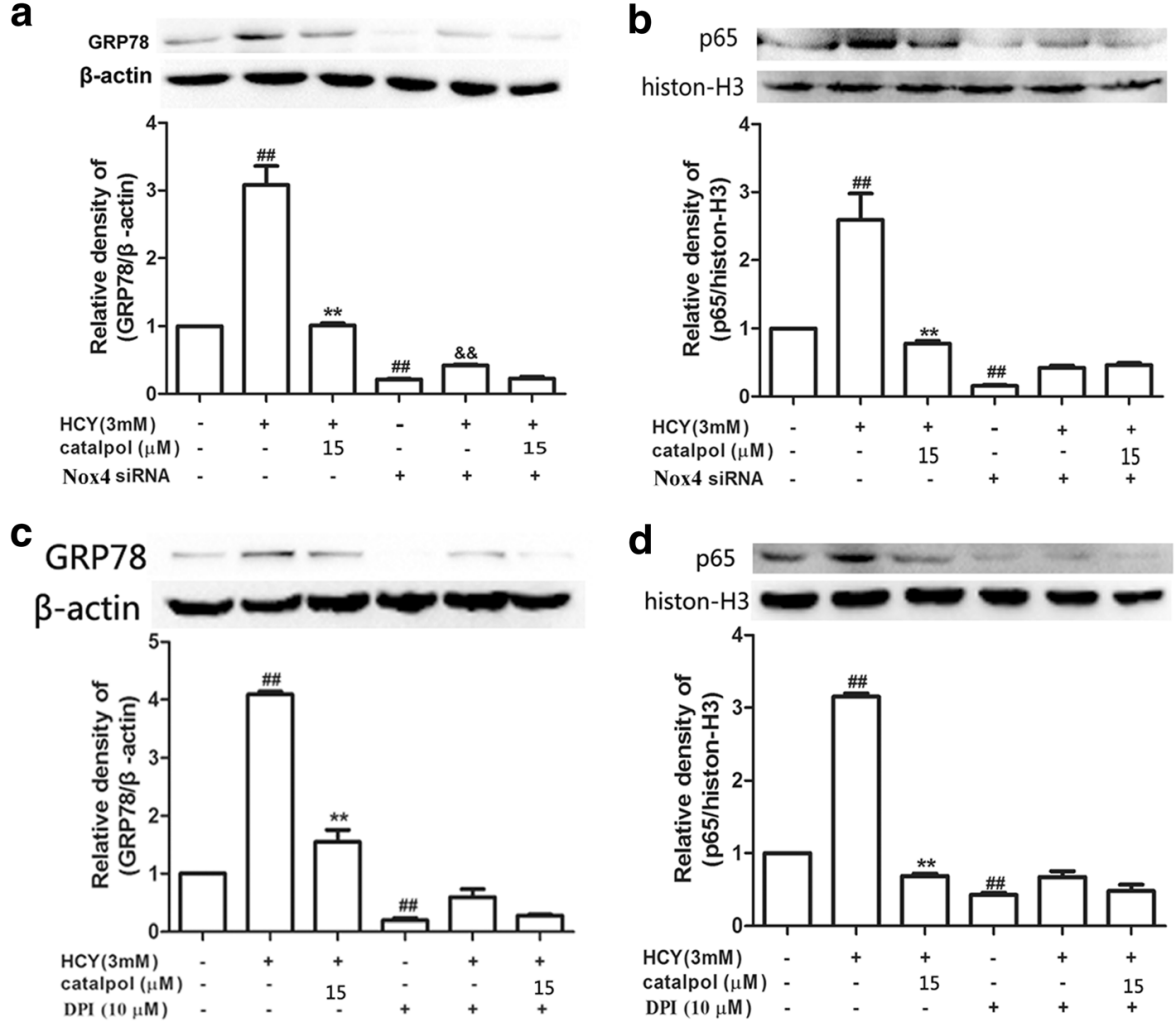
The effects of catalpol on HCY-induced GRP78 and p65 activations and the effect is associated with inhibiting Nox4. Cells were treated with Nox4 siRNA (50 nM) and 10 μΜ DPI. Western blot analysis was performed using anti-GRP787, anti-p65 antibodies. (**a**) Effect of catalpol on GRP78 protein expression level treated with Nox4 siRNA. (**b**) Effect of catalpol on p65 protein expression level treated with Nox4 siRNA. (**c**) Effect of catalpol on GRP78 protein expression level treated with DPI. (**d**) Effect of catalpol on p65 protein expression level treated with DPI. Results represent the mean ± S.D. from three independent experiments. ^*##*^*p* < 0.01 *vs.* control, ^**^p < 0.01 *vs.* HCY, ^&&^*p <* 0.01 *vs.* Nox4 siRNA.

In order to investigate whether the reduction of the expressions of NF-κB and ER stress-associated proteins by catalpol was involved in the inhibition of Nox4, we employed Nox4 siRNA to evaluate the related protein expressions in HAECs by Western blotting. As shown in Fig. 10a, b, in normal cells, catalpol treatment significantly decreased the expressions of NF-κB/p65 and GRP78 compared to the HCY-induced-alone group, and the decrements of NF-κB/p65 and GRP78 were in 3.03-fold and 3.07-fold respectively. However, in Nox4 siRNA cells, the expressions were reduced in 1.84-fold and 1.09-fold only. Simultaneously, we also used the selective cell-permeable Nox4 inhibitor, DPI to confirm the results. As demonstrated in Fig. 10c, d, in normal cells, the inhibiting effects of catalpol on NF-κB/p65 and GRP78 protein expressions were 2.64-fold and 4.6-fold, respectively. However, in the presence of DPI, the expressions of the proteins for NF-κB/p65 and GRP78 were reduced only by 2.17-fold and 1.4-fold, respectively. These results indicated that the inhibiting effects of catalpol on NF-κB/p65 and GRP78 protein expressions in the cells in which Nox4 was knocked down were less than that in the normal cells. Taking these results together, catalpol could inhibit activation of NF-κB signal pathway as well as over-expression of GRP78 induced by HCY partly *via* inhibiting the expression of Nox4.

### Catalpol Alleviated HCY-Induced over-Expression of Nox4 and Activation of ER Stress *via* Blocking NF-κB/p65 in HAECs

**Fig. 11:**
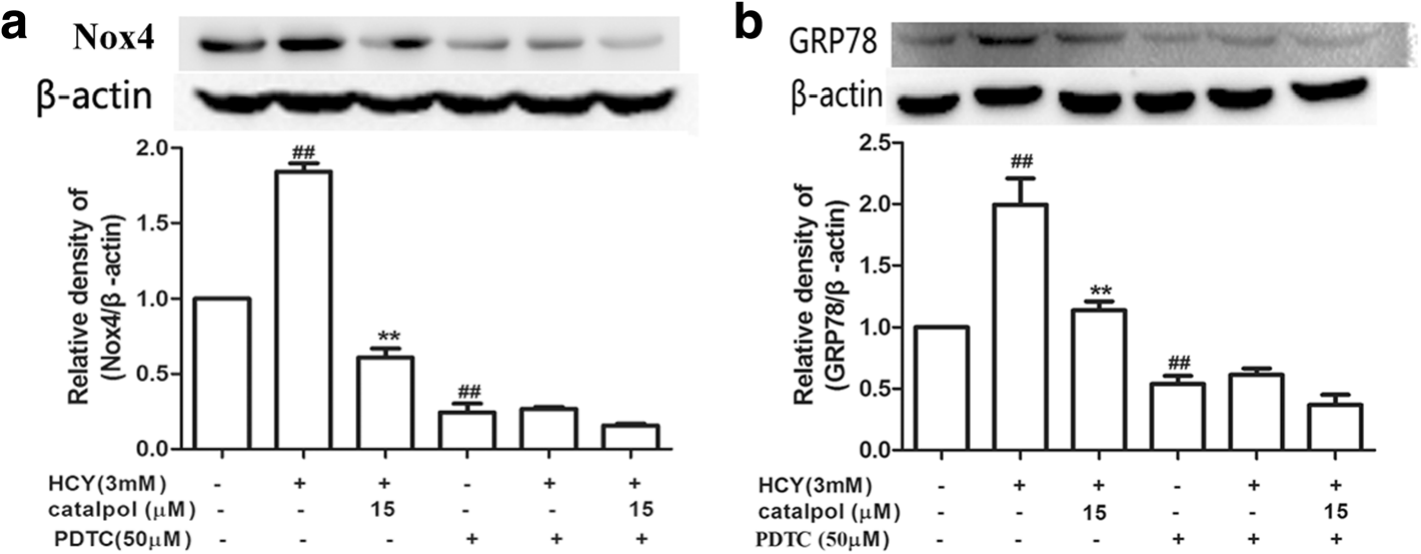
The effects of catalpol on HCY-induced Nox4 and GRP78 expression and the effect is associated with inhibiting NF-κB pathway. Cells were treated with 50 μΜ PDTC. Western blot analysis was performed using anti-Nox4, anti-GRP78 antibodies. (**a**) Effect of catalpol on Nox4 protein expression level. (**b**) Effect of catalpol on GRP78 protein expression level. Results represent the mean ± S.D. from three independent experiments. ^*##*^*p* < 0.01 *vs.* control, ^**^p < 0.01 *vs.* HCY.

For the sake of further identifying whether catalpol protected HAECs from oxidative stress and ER stress induced by HCY was involved in blocking NF-κB/p65, the protein expressions of Nox4 and GRP78 were measured by Western blotting after NF-κB signal pathway blocker PDTC was adopted. As shown in Fig. 11a, b, in normal cells, the protein expressions were increased in HCY-induced group compared to control group. Catalpol resulted in 2.95- and 1.75-fold decline compared to HCY group. However, in NF-κB/p65 blocked-down cells, catalpol reduced Nox4 and GRP78 protein expressions in only 1.74 and 1.66-fold, respectively, compared to HCY treatment alone group. It suggested that catalpol reduced oxidation and ER stress in HCY-treated HAECs partly through inhibiting NF-κB signal pathway.

### Catalpol Relieved HCY-Induced ROS over-Generation and the Protein over-Expressions of Nox4 and NF-κB/p65 through Inhibiting ER Stress in HAECs

**Fig. 12:**
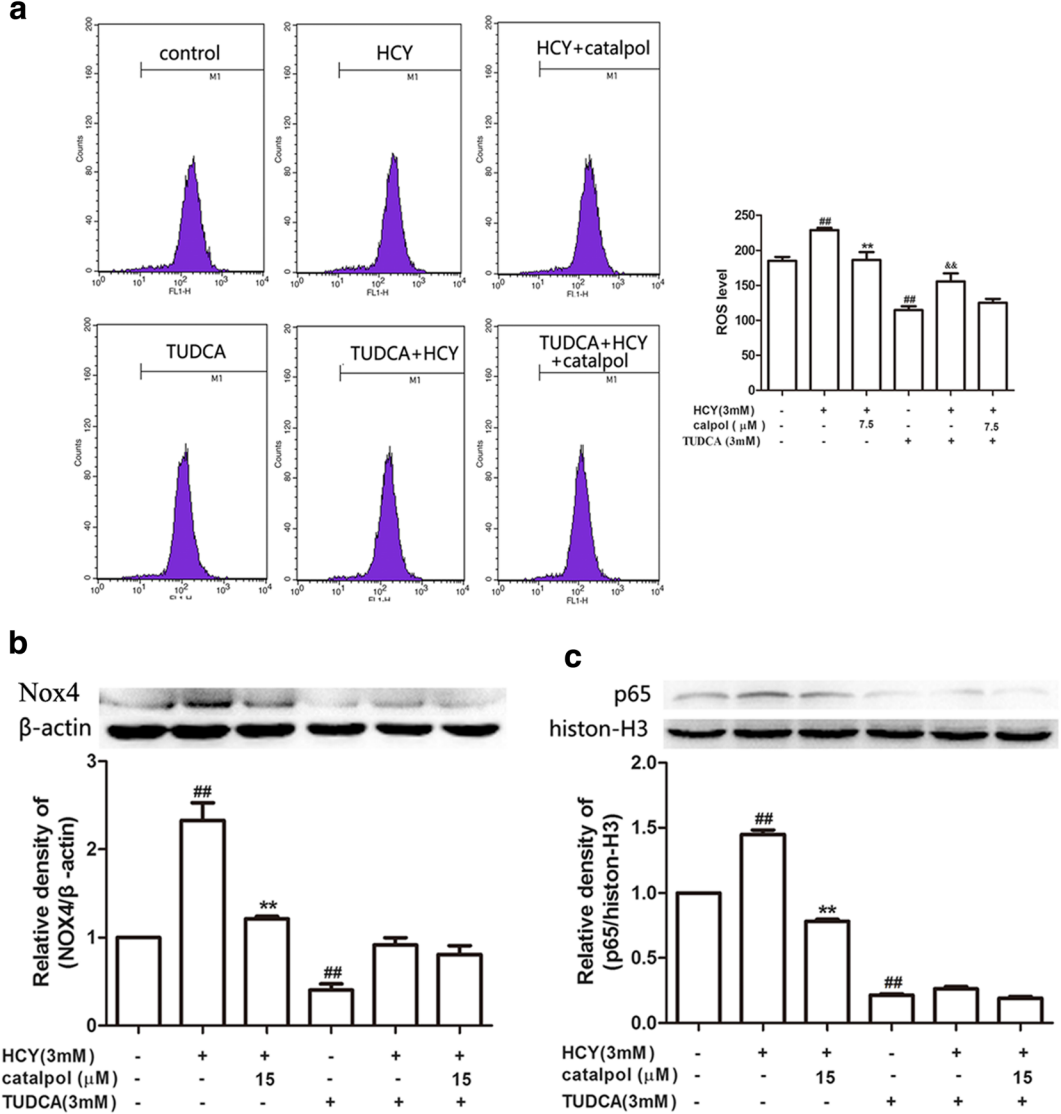
The interaction among NF-κB, Nox4/ROS and ER stress in HAECs. Cells were treated with 3mΜ TUDCA. (**a**) The intracelluar ROS production. Western blot analysis was performed using anti-Nox4, anti-p65 antibodies. (**b**) Effect of catalpol on Nox4 protein expression level. (**c**) Effect of catalpol on p65 protein expression level. Results represent the mean ± S.D. from three independent experiments. ^*##*^*p* < 0.01 *vs.* control, ^**^p < 0.01 *vs.* HCY, ^&&^*p <* 0.01 *vs.* Nox4 siRNA.

It has been reported that Nox4/ROS-NF-κB contributes to the progression of ER stress and oxidation [29, 30]. To further estimate the interaction among ER stress, oxidative stress and NF-κB/p65 signaling pathway, we used ER stress-specific inhibitor, TUDCA, to test the alteration. As the results showed in Fig. 12a, catalpol could decrease ROS generation induced by HCY in normal cells significantly. However, in TUDCA-treated cells, catalpol had no significant reducing effect on ROS over-generation induced by HCY. As shown in Fig. 12b, c, in normal cells, catalpol significantly attenuated HCY induced Nox4 and NF-κB/p65 protein over-expressions in 1.92- and 1.85-fold, respectively. Moreover, in TUDCA treatment cells, catalpol resulted in only 1.13- and 1.42-fold decline in suppressing Nox4 and NF-κB/p65 protein over-expressions induced by HCY. That is to say the extents of these decreases caused by catalpol in TUDCA treatment cells were lower than that in normal cells. Collectively, ER stress mediated HCY-stimulated intracellular ROS generation and catalpol partly ameliorated Nox4 and NF-κB/p65 protein expressions in HCY-induced cells *via* blocking ER stress possibly.

## DISCUSSION

Homocysteine is an anechogenic sulfurated amino acid, which is rooted in ingested methionine including eggs, cheese, fish, meat, and poultry [31]. Hyperhomocysteinemia is a pathological process characterized by an elevation in plasma concentration of total homocysteine [32]. Recent studies observed that HHCY is an indicator of adverse cardiovascular events among the patients of ischemic heart disease and stroke [33, 34]. Homocysteine could cause oxidative stress and ER stress, leading to inflammatory response, growth arrest, and programmed cell death in cultured human vascular endothelial cells.

It is well known that HCY yields superoxide and hydrogen peroxidase by auto-oxidation to trigger oxidative stress to blood vessels [35]. The present study observed that HCY inhibited the generation of glutathione peroxidase and enhanced intracellular production of superoxide, including MDA level and LDH release. Given that catalpol could activate the expression of anti-oxidant enzymes, catalpol could preliminarily prevent atherogenesis by inhibiting oxidative stress. NAD (P) H oxidases are the major sources of intracellular ROS production in Hcy-induced endothelial cells and NAD (P) H oxidases consist of multiple subunits, containing membrane-associated Nox1–4 and p22^phox^ subunits, with Nox4 playing the key role in vasculopathies [36, 37]. In this study, we found that Hcy-induced Nox4 and p22^phox^ over-expressions were both down-regulated by catalpol. Catalpol also enhanced the superoxide anion scavenging ROS capacity and reduced endothelial cell injury. This provided that catalpol could protect against the oxidative damage of cytotoxic oxygen radical and improve the function of cardiovascular system.

Evidence has shown that an excess of HCY could induce accumulation of misfolded proteins and trigger the ER stress [38]. GRP78 is the typical ER chaperone protein that binds to ATF6, PERK, and IRE1α in normal condition. When the cells are under stress, these transmembrane transducers will be disaggregated from GRP78 and activate down-stream signaling genes to initiate UPR [39]. Our results showed that the expressions of ER stress-associated proteins including GRP78, ATF6, IRE1α, and PERK phosphorylation were increased in the presence of HCY. Catalpol pre-treatment significantly alleviated the over-expressions of ER stress-related proteins and recovered the homeostasis. In addition, previous studies have reported that ER stress and oxidative stress are relevant [30]. This finding is consistent with our results showing that both Nox4 and ER stress-specific inhibitors (DPI and TUDCA) suppressed the effects of catalpol on inhibiting oxidative stress and the expression level of NF-κB/p65.

A progressive inflammatory response is the major contributor to atherosclerosis, and NF-κB, as a pro-inflammatory factor, involved in atherosclerotic lesion from cardiovascular disease [40, 41]. The activation of NF-κB pathway induced by HCY generally increased the expression of cytokines, chemokines, and cellular adhesion molecules [42]. In the present study, we investigated that HCY stimulated the activation of NF-κB pathway, the expressions of MCP-1, VCAM-1, and ICAM-1, leading to inflammatory reaction in cultured endothelial cells. Catalpol can exert its anti-inflammatory effects *via* multiple routes, including suppression of the overproduction of focal inflammatory mediators, and the protection of tissues by mediating cytokines. In our present study, catalpol exerted its anti-inflammatory property through reducing the expressions of pro-inflammatory factors. Many studies have verified that the translocation of NF-κB is associated with programmed cell death *via* activating caspase family [43]. Indeed, our results suggested that catalpol relieved the cleaved caspase-3, caspase-9, and bax expression, as well as elevated the expression of bcl-2. Taking together, the effects of catalpol might be achieved by maintaining endothelial function *via* reducing oxidative stress and ER stress and inflammation.

In conclusion, these results indicated that HCY could cause endothelial impairment and mitochondrial dysfunction in HAECs, including decreased membrane mitochondrial potential and increased oxidative stress, ER stress as well as inflammation. Catalpol pre-treatment significantly alleviated oxidative stress, inflammatory response and maintained the ER and mitochondrial integrity in HCY-induced HAECs. Consequently, the actions of catalpol protecting against HCY-induced injuries in HAECs by inhibiting Nox4/ROS-NF-κB pathway and ER stress might be used in the treatment and/or prevention of atherosclerosis. Catalpol would be a potentially novel drug for the treatment and prevention of AS.

## CONCLUSION

Catalpol can ameliorate HCY-induced ER stress and oxidation then reduced cells apoptosis and inflammation in HAECs. These results suggested catalpol played a key role in protecting endothelial dysfunction in HHCY-induced cardiovascular diseases.
